# Current and future technology for minimally invasive ablation of renal cell carcinoma

**DOI:** 10.4103/0970-1591.70584

**Published:** 2010

**Authors:** Branden G. Duffey, J. Kyle Anderson

**Affiliations:** Department of Urology, University of Minnesota

**Keywords:** Kidney, kidney neoplasm, kidney cancer

## Abstract

**Purpose of Review:**

To provide an overview of the technologic advancements in the field of ablative therapy, focusing on the treatment of renal neoplasms.

**Materials and Methods:**

A MEDLINE search was performed using each specific ablative technique name as the search term. Articles written in the English language were selected for review. In cases of multiple reports by a single institution, the most recent report was utilized. Pertinent articles specific to the technologic advancement in ablative therapy were selected for review.

**Recent Findings:**

Intermediate-term oncologic outcomes of radiofrequency ablation (RFA) and cryoablation (CA) for the treatment of small renal masses are encouraging. For thermal therapies, molecular adjuvants to enhance cellular kill and local control have been developed. Improvements in microwave technology have allowed for reductions in antenna size and increases in ablation size. Laparoscopic high-intensity focused ultrasound (HIFU) probes have been developed to overcome the limitations of transcutaneous energy delivery, but HIFU remains experimental for the treatment of renal lesions. Irreversible electroporation (IRE), a novel nonthermal ablative technique, is currently undergoing clinical investigation in human subjects. Histotripsy causes mechanical destruction of targeted tissue and shows promise in treating renal and prostate pathology.

**Summary:**

Ablative techniques are commonly utilized in the primary treatment of urologic malignancies. The purpose of this review is to discuss technologic advances in ablative therapies with emphasis on the treatment of renal masses. RFA and CA show acceptable intermediate-term efficacy and technical refinement continues. Emerging technologies, including microwave thermotherapy, IRE, HIFU and histotripsy, are described with emphasis on the mechanism of cellular kill, energy delivery, and stage in clinical development.

## INTRODUCTION

Ablative technologies have been embraced as primary treatment options for several malignancies, including prostate, testicular, hepatic, lung, and renal cancer. Increased utilization of computed tomography imaging has accompanied a rise in the diagnosis of renal masses, many smaller than 4 cm.[1,2] A substantial proportion of these tumors, that is, 20–30%, will be benign.[3,4] Partial nephrectomy, the gold-standard treatment for small renal masses (SRMs), is associated with significant morbidity and may not be an acceptable treatment for every patient, especially those with significant comorbidities. Ablative therapies have been developed as an attempt to provide acceptable oncologic control, while reducing the morbidity associated with partial nephrectomy.

The purpose of this review is to discuss the significant technologic and clinical developments in the field of ablative therapy for the treatment of SRMs. Cryoablation (CA) and radiofrequency ablation (RFA), the most common ablative techniques used for the ablation of SRMs, have been reviewed with emphasis on clinical outcomes and the use of molecular adjuvants. Newer ablative therapies are described in this article, with focus on the energy type, generator, delivery system, and stage in clinical development. The sources utilized in this review were identified during a MEDLINE database review. The name of each ablative technique was used as the search term. Pertinent articles focusing on technologic development and published in English were selected for review. In the case of multiple publications from the same institution, the most recent publication is presented unless there is significant clinical relevance to older publications.

## RADIOFREQUENCY ABLATION

RFA causes hyperthermic destruction of targeted tissue. RFA probes are designed to deliver a continuous high-frequency electrical current to the surrounding tissue through the noninsulated distal portion of an electrode. This energy causes resistive friction in the surrounding tissues that subsequently generates heat.[5,6] The effects of temperatures exceeding 50°C are multiple and varied, causing chromosomal alterations, protein denaturation, damaging cellular membranes and associated transport proteins, and microvascular and arteriolar occlusion.[7,8] Temperatures >100-105°C result in tissue boiling, vaporization, and carbonization, resulting in periprobe char that decreases effective energy transmission and may result in suboptimal ablation.[9] Although general consensus has held that temperatures should reach 50–100°C throughout the targeted volume, successful ablation depends not only on the temperature attained, but also on the duration that temperature is held. Time–temperature points resulting in >99% cell death in an in vitro renal cell study were 55°C for 30 min, 60°C for 10 min, 65°C for 8 min, and 70°C for 1 min.[10] In vivo the temperature necessary to cause cell death may further be lowered by tissue ischemia, pH changes, and inflammatory response.

Radiofrequency energy is delivered via probes powered by either temperature- or impedance-based generators. Temperature-based systems (RITA 1500X, Angiodynamics, Queensbury, NY) deliver energy until a specific temperature has been reached for a predetermined period of time. Target temperatures are limited to 100–105°C to avoid tissue char and incomplete ablation. Impedance-based systems (RF 3000, Boston Scientific, Natick, MA, and Cool Tip, ValleyLab, Boulder, CO) conclude treatment when the periprobe tissue reaches a predefined impedance signifying tissue desiccation such that electrical current passage is impaired.

A wide variety of RFA probes are available and the probe selected depends on the surgical approach, lesion size, location, and imaging system utilized. Single-needle probes are well suited for treating small lesions or may be grouped into a cluster for the treatment of larger lesions. Multitined probes are useful in treating larger lesions, deploying several electrodes from the distal tip reaching diameters up to 7 cm. Bipolar devices (Habib 4X, Angiodynamics, Queensbury, NY) are available for both open and laparoscopic applications and have been utilized to create an avascular tissue plane facilitating clampless laparoscopic and robotic-assisted partial nephrectomy.[11,12] Multiple-electrodes ablation systems (Cool Tip, ValleyLab, Boulder, CO), designed to create larger consistent lesions, utilize a generator and switching controller to drive up to 3 independent electrodes, switching among them when impedance increases 30 Ω greater than baseline or after an interval of 30 s, whichever occurs first.[13]

As clinical experience with RFA has grown, short- and intermediate-term results of SRMs treated with RFA have been published and are encouraging. Contemporary oncologic outcomes from high-volume centers report recurrence-free rates of 90–96.8 % following RFA in patients with mean ages and tumor volumes of 63.6–70.4 years and 2.0–3.2 cm, respectively.[14–17] Two recent meta-analyses evaluating the treatment of SRMs reported slightly higher rates of local recurrence following RFA (11.7–12.9%) compared with partial nephrectomy (2.6%) and CA (4.6–5.2%), but observed a low incidence of metastatic disease (0.9–2.5%), rates similar to CA, partial nephrectomy, and surveillance.[4,18] Additionally, univariate and multivariate analyses suggest that RFA carries a greater risk of local recurrence and the need for retreatment compared with the other management options.[4,18] When interpreting the outcomes of RFA, it is important to note that definitions of local recurrence and treatment failure are not standardized, making it difficult to differentiate an incomplete ablation (which could be treated by repeat ablation and has little effect on prognosis) and a true local recurrence. Additionally, a percutaneous approach has been most frequently utilized for RFA and patients tend to be older, suggesting a significant selection bias. A percutaneous approach offers the potential of limited morbidity, especially in patients with significant coexisting diseases. In select patients, the decreased risk associated with percutaneous RFA may outweigh the potential morbidity of a more invasive treatment (ie, laparoscopic-assisted ablation) making this a viable option. Overall, RFA is an oncologically sound approach for the treatment of SRMs, however, superior local control rates seen with CA (more frequently delivered via a laparoscopic approach) suggest that improvements in technique may improve outcomes. Recent work has suggested that percutaneous RFA under general anesthesia with contrast-enhanced imaging (compared with conscious sedation with varied imaging modalities) or laparoscopic-guided probe placement with deployment of multiple thermosensors may offer improved probe placement and local control; however, these suppositions have not been evaluated in randomized studies.[19,20] In select patients, both CA and RFA are clinically proven techniques with acceptable oncologic efficacy and are useful options for the treatment of SRMs.

Histologic examination of SRMs treated with RFA indicate that local failure results from “skip” lesions within the tumor and inadequate treatment at the periphery.[21,22] Assuming adequate probe placement, these failures probably arise from either heterogenous tumor tissue characteristics leading to unpredictable current flow (“skip” lesions) or peritumor vasculature creating a heat sink effect (temperatures at margin not sufficient for cellular kill). The common strategies to prevent these negative outcomes have included extending the treatment zone 5–10 mm past the tumor margin, performing multiple ablations of the same tumor by varying the probe angle or rotating the array in the case of multitined probes, and avoiding ablative treatment in tumors in close proximity to the renal hilum. More recently, the administration of specific drugs, functioning as RFA molecular adjuvants, has been evaluated in an effort to increase treatment efficacy, decrease the rates of local failure, decrease the time and extent of treatment, and spare healthy adjacent tissue. In one study, liposomal-packaged chemotherapeutics administered with RFA were found to increase ablation volumes 39–61% compared with RFA alone.[23] Adjuvant arsenic trioxide prior to RFA in a VX2 rabbit model resulted in reduced tumor blood flow and increased ablation volumes by 83% compared with controls, effects probably caused by apoptosis, vascular shutdown, and thermal sensitizing effects.[24] Of the agents evaluated thus far, the most clinically relevant is probably sorafenib. This vascular endothelial growth factor and platelet-derived growth factor receptor inhibitor, and anti–RAF kinase agent decreased microvascular density and increased ablation size from 6.7 ± 0.7 mm to 11.1 ± 0.3 mm (*P* < 0.01) compared with control animals.[25] Recently, sorafenib was used to reduce tumor size from 3.7 to 1.7 cm in a single functioning kidney facilitating RFA, resulting in successful local control after repeat ablation.[26] Molecular adjuvant administration may improve local control and spare healthy adjacent tissue by increasing the susceptibility of the tumor to thermal injury; however, further research is necessary prior to clinical application.

## CRYOABLATION

CA is a thermal ablative technique designed to remove heat from tissue, resulting in temperatures ≤ –40°C and ice ball formation encompassing the targeted tissue with subsequent hypothermic stress and cellular death.[27] During CA, cellular injury occurs via several mechanisms, which can be broadly classified as cellular, vascular, or immunologic. Direct cellular injury occurs from the formation of ice in the extracellular and intracellular space. As ice forms in the extracellular space, osmolarity perturbations cause an efflux of water into the extracellular space leading to intracellular hypertonicity, altered pH, and protein denaturation. Additionally, ice formation causes mechanical disruption of cellular membranes. Vascular injury occurs as ice crystals propagate along the walls of blood vessels causing mechanical disruption, microvascular shutdown, and ischemia. Delayed cellular death may be mediated via immunologic mechanisms stimulated by the release of tumor antigens or apoptosis from cells at the periphery of the cryolesion (temperatures > –40°C).[28,29]

Modern renal CA devices utilize the rapid cooling of argon gas as it passes through specially constructed cryoprobes (the Joule–Thompson effect). The probes are capable of creating ice balls of widely varied sizes (31 × 36 mm to 45 × 64 mm); however, the –40°C isotherm is generally quite smaller (11 × 19 mm to 22 × 44 mm). CA probe diameters range from 1.2 to 3.8 mm and are available in various lengths, configurations, and some offer magnetic resonance imaging (MRI) compatibility. Galil Medical (Yokneam, Israel) and Endocare (Irvine, California, USA) currently manufacture CA systems designed for renal ablation. Surgical approaches to renal CA have included open, percutaneous, laparoscopic, natural orifice translumenal endoscopic surgery, and laparoendoscopic single site.[30,31]

Intermediate oncologic outcomes indicate that CA is a curative option for the treatment of SRMs in patients unfit or unwilling to undergo partial nephrectomy. Larger series with follow-up ranging from 9 to 36 months report excellent local control (95–100%) and cancer-specific survival (95–100%) in patients with single sporadic renal masses.[32–37] Aron *et al*. recently reported the oncologic outcomes of 88 patients treated with laparoscopic CA and a minimum follow-up of 5 years.[38] In 82 patients with a sporadic single renal mass followed for a mean of 83 months (range 60–120 months) 5-year overall survival, cancer-specific survival, and recurrence-free survival was 83%, 95%, and 78%, respectively. Additionally, the estimated 10-year Kaplan–Meier overall survival, cancer-specific survival, and recurrence-free survival was 57%, 88%, and 51%, respectively. Kunlke’s meta-analysis evaluating nephron sparing techniques in the treatment of SRMs found that CA carries a higher risk of local recurrence (RR = 7.45) compared with partial nephrectomy, but no difference in the development of metastatic disease.[4] When comparing CA and RFA, CA was associated with lower incidence of local recurrence (5.2% vs 12.9%, *P* < 0.001) and need for retreatment (1.3% vs 8.5%, *P* < 0.001), but similar rates of metastatic disease (1% vs 2.5%, *P* = 0.06).[18] Lesions treated with CA in this meta-analysis were approached laparoscopically most often (65%), whereas 94% of RFA treatments were delivered percutaneously, raising the question whether the surgical approach alters the need for retreatment and possibly the oncologic outcome. A recent single institution retrospective analysis of the efficacy and complications of laparoscopic CA for larger renal masses found no difference in short-term local control in tumors < 3.0 cm (n = 30, mean tumor size = 1.8 cm, range 0.7–3.0 cm) vs tumors > 3.0 cm (n = 21, mean tumor size = 4.0 cm, range 3.1–7.5 cm); however, the treatment of tumors > 3.0 cm was accompanied by more complications (62% vs 0%), need for blood transfusion (38% vs 0%), and longer hospitalization (3.52 vs 1.65 days).[34]

As previously mentioned, cryoinjury is mediated by vascular, immunologic, and direct cellular effects. Although instant cell death is achieved within the –40°C isotherm, cell death at the edges of the iceball, where temperatures range from –40 to –0.5°C, is uncertain. Molecular adjuvants of CA have been administered in an effort to augment the vascular, immunologic, and direct cellular effects of cryoinjury, to make tissue at the ice ball edge more susceptible to injury, and to potentially spare normal healthy adjacent tissue. Thermophysical adjuvants, such as antifreeze proteins, salts, and some amino acids modify the crystalline ice during freezing, causing additional direct cell injury due to the presence of ice crystals. These adjuvants have been utilized in human and rat prostate models, but clinical success is limited by the need to successfully target specific tissues with therapeutic doses of adjuvant, while minimizing toxicity to other tissues.[39,40] Coadministration of chemotherapeutic agents with CA has been explored in several models as an alternative method of enhancing the direct cellular effects of cryoinjury. Multiple in vitro studies have shown the ability of chemotherapeutics, such as 5-fluorouracil, cisplatin, and bleomycin to augment cell death at milder freezing conditions (ie, between –5 and –15°C); however, no study to date has shown augmentation of cell kill to the edge of the iceball.[41–45] Although this approach appears to be promising, most experimental work has been in vitro, and more in vivo evidence is needed to address the issues of exact mechanism of injury, dose, timing, and drug selection.[27] Exacerbation of the adverse vascular effects of CA has been rigorously studied, but only TNF-α has been shown to augment cell death up to the edge of the ice ball. TNF-α is associated with multiple vascular and immunologic events, including endothelial cell apoptosis, increased procoagulant activity, decreased anticoagulant activity, increased inflammatory cell response and the production of other cytokines.[46–48] The major barrier to clinical use of TNF-α has been the significant side effects associated with systemic administration of doses required to achieve a local effect. Novel delivery methods are currently being investigated and in a recent study, TNF-α was delivered via a gold nanoparticle, which augmented the ablative process and greatly reduced systemic side effects.[49]

## MICROWAVE

Microwaves lie on the electromagnetic spectrum between infrared and radiowaves with frequencies ranging from 900 to 2450 MHz. Microwaves ablate targeted tissue by agitating water molecules, producing friction and heat, ultimately inducing cell death via coagulative necrosis.[50] The benefits of microwave thermotherapy include high intratumoral temperature (approaching 150°C), reliance on electromagnetic energy that does not require conduction through tissue eliminating the need for grounding pads and minimizing the effects of tissue desiccation and char, rapid heat generation making it less susceptible to heat sink from large vascular structures, and multiple antennas may be utilized simultaneously.[51,52] The drawbacks of microwave thermotherapy, including limited zone of ablation, large antenna size, significant retrograde heating of the delivery antenna, and the need for a microwave generator for each antenna utilized have blunted its widespread acceptance.

Refinements in antenna design have focused on reducing size, maximizing energy delivery to the target tissue, and minimizing retrograde heat propagation along the antenna, and thus reducing the risk of cutaneous burns. Changes in generator design and power distribution have permitted multiple antennas to be powered by a single generator.

Clark *et al*. ablated 10 renal masses using 13-gauge saline cooled antennas with improved material and structural properties designed to improve energy deposition into the tissue.[52] Single-probe configuration achieved a mean ablation size of 4.1 × 2.7 × 2.2 cm, whereas a 3-probe configuration averaged 5.7 × 4.7 × 3.8 cm. The only cutaneous complication was a grade I skin burn at an antenna, which was not connected to the cooling system. This system is no longer available and has been replaced by the Evident™ MW Ablation System (ValleyLab, Boulder, CO). Probes with the Evident™ system are designed for either percutaneous or open ablative use. Percutaneous antennas are 13-gauge and internally cooled with circulated saline, whereas the antennas used during open ablation are 11-gauge and have a copper choke to minimize back heating. The Evident™ MW ablation system has been currently approved by the US Food and Drug Administration for use in the hepatic tumors with approval for renal neoplasms underway.

Liang *et al*. reported intermediate-term results of percutaneous ultrasound-guided microwave ablation of 12 renal masses < 4 cm showing complete ablation in a single session without evidence of tumor regrowth at a mean of 10.8 months.[53] They utilized the KY2000 MW ablation system (Kangyou Medical Instruments, Nanjing, People’s Republic of China). The generator can produce up to 100 W and drive 2 antennas simultaneously. The antennas are 15-gauge, polytetrafluoroethylene-coated, and saline cooled. This system is also equipped with a thermal monitoring system that can measure temperature during ablation.

Brace *et al*. further reduced antenna size by constructing a triaxial 17-guage antenna.[51] The size reduction and changes in design both reduce the local traumatic effects of antenna insertion and enables tuning of the antenna for a specific tissue type and frequency by adjusting the active length and insertion depth.[54] A 2.45-GHz generator (Cober Muegge, Norwalk, CT) capable of continuously supplying 300 W may be coupled with a 2- or 3-way power splitter (SM Electronics, Fairview, TX) permitting simultaneous activation of up to three 17-gauge triaxial antennas with a single generator.[55] A comparison of microwave (single- vs multiple triaxial antennas) vs radiofrequency (single- vs 3-electrode array) in a canine model revealed 3-electrode radiofrequency and single-antenna microwave ablation zones were significantly larger than single-electrode RF zones. Although there were no differences between single microwave and multiple RF ablation zones, tissue temperatures were higher during microwave ablation (maximum temperature of 123 vs 100°C for RF).[55]

## HIGH-INTENSITY FOCUSED ULTRASOUND

High-intensity focused ultrasound (HIFU) focuses ultrasound waves that propagate through normal tissue and converge on targeted tissue. At the focal zone, ultrasound energy is converted to heat resulting in protein denaturation and coagulative necrosis.[56] Focal zone temperatures during HIFU quickly exceed 80°C during treatment.[57] To create a clearly demarcated lesion, the power density should exceed 100 W/cm, a value sufficient to produce temperatures ≥ 65°C within a pulse duration of <5 s.[56]

Extracorporeal HIFU systems currently employed for treatment of SRMs utilize 0.6–1.8 MHz piezoelectric transducers driven by generators capable of delivering up to 2000 W.[58] When using a 1 MHz transducer, ultrasonic energy is typically delivered in at least 15-s intervals with a pulse duration of 4–6 s.[56,59] The focal zone size varies with transducer frequency and focal length, but generally ranges from 3–4 mm × 12–32 mm.[56,59,60] In the Storz Investigational HIFU device (Storz, Germany) ultrasound waves delivered by a hand-held or mechanically controlled transducer are coupled to the patient’s body through a polyurethane cushion filled with degassed water. In the Chongquing “HAIFU” device (Chongquing, China), the patient lies on a treatment table and the transducer is located within a basin filled with degassed water to couple the ultrasonic energy to the patient. In both the systems, the treated area is monitored with a confocally mounted 3.5 MHz B-mode ultrasound transducer.

To date, studies evaluating transcutaneous renal HIFU have been disappointing. Targeted renal tissue is inconsistently ablated and the ablated volume frequently is smaller than the planned treatment area.[56,59,61] These unsatisfactory results are mainly due to complex acoustical interfaces surrounding the kidney (ie, ribs and bowel) and mobility of the kidney. Target movement during energy delivery may decrease the time that ultrasonic energy is delivered to a specific area resulting in failure to reach time–temperature combinations necessary for cell death. Difficulties with target motion could theoretically be solved using multichannel focused ultrasonic systems and multiprobe systems of small-aperture confocal HIFU transducers. These solutions have been evaluated experimentally, but have not undergone clinical evaluation. Additionally, there is a lack of real-time monitoring of the HIFU process, as standard thermocouples interfere with ultrasonic energy and cannot be utilized, and MRI thermography requires a near motionless target.

Laparoscopic HIFU was developed to circumvent the difficulties associated with the transcutaneous approach. Paterson *et al*. evaluated laparoscopic renal HIFU using a modified transducer (frequency 4 MHz, focal length 30 mm) and probe (18 × 30 mm) (Sonablate 200, Focus Surgery, Indianapolis, IN). The average treatment duration was 18.3 min and the ablated lesions matched the planned lesion size (21 × 17 × 11 mm and 21 × 17 × 10 mm, respectively).[62] A phase I study in human subjects using a modified laparoscopic probe (Sonatherm, Misonix Inc, Farmingdale, NY) suggested that laparoscopic renal HIFU is feasible and allows sufficient tumor destruction.[63] Performed in a “continuous ON” mode under computer control, mean ablated size was 10.2 cm^3^ with a mean ablation time of 19 min. Of 7 tumors ablated and removed after HIFU, 4 showed complete ablation of the entire tumor. Two tumors had a 1- to 3-mm rim of viable tissue immediately adjacent to where the HIFU probe was placed, indicating the need to keep the transducer > 7 mm away from the tumor. One tumor showed a central area with about 20% vital tissue.

Currently, HIFU of renal tumors must be considered experimental. Transcutaneous HIFU does not currently permit successful tumor destruction and is not considered an alternative to surgical excision. Laparoscopic HIFU has the potential to overcome the limitations associated with transcutaneous treatment, but further studies evaluating oncologic efficacy are necessary.

## IRREVERSIBLE ELECTROPORATION

Irreversible electroporation (IRE) is a newly developed nonthermal tissue ablation technique in which intense short-duration electrical fields are used to permanently permeabilize the cell membrane, presumably through the formation of nanoscale defects in the cell membrane.[64,65] Depending of the resultant transmembrane electrical potential, the application of an electrical pulse can (1) have no effect on the cell; (2) reversibly open cellular membranes after which the cells survive; or (3) irreversibly open the cell membrane leading to cell death. Although the exact mechanism of IRE is unknown, it is thought that when the potential drop across the membrane exceeds approximately 1 V, permanent structural rearrangement of the lipid bilayer occurs, creating aqueous pathways or pores for ions and macromolecules to pass through.[66] The irreversible permeabilization of cell membrane leads to changes in cell homeostasis and cell death.

Because of its unique mechanism of ablation, IRE has several advantages when compared with thermal-based ablative techniques. Because IRE does not rely on thermal energy, the targeted tissues adjacent to large vascular structures are not affected by a “heat sink” effect.[67] IRE destroys the cellular components of a tissue, but does not affect the underlying collagen network of tissue, thereby preserving the basic tissue structure. Indeed, deliberate treatment of the rectum and neurovascular bundles in a canine prostate protocol showed sparing of the neurovascular bundles and no evidence of rectal injury or fistula formation.[68] Additionally, sparing of the tissue scaffolding and arteriolar vasculature may facilitate healing and rapid radiologic resolution. Treatment times with IRE are shorter than conventional thermal ablative techniques (8.4 ± 1.8 min) and generate lesions of comparative size (3.2 × 2.5 × 3.9 cm). Lastly, tissues treated with IRE have a sharp line of demarcation between ablated and nonablated areas, facilitating pathologic evaluation.[67,68]

The energy required to accomplish IRE is delivered by 15 cm monopolar (18-gauge) or bipolar (16-gauge) electrodes. The distal 4 cm of each electrode is uninsulated. The electrodes are connected to a high voltage generator capable of delivering 1000–3000 V per pulse and controlled via a graphical user interface (Angiodynamics, Queensbury, NY). Prior to the procedure, the operator sets the desired pulse number, pulse duration, repetition rate, and voltage.

IRE can be performed as a real-time ultrasound-guided intervention as no hyperechoic gas is generated during ablation. Lee *et al*. reported that during IRE treatment, a spherical hypoechoic area of ablation is detected during and immediately after IRE in ultrasound images.[67] They speculate the hypoechogenicity is due to increased intra-/extracellular water molecules after opening of transmembrane pores by the high voltage of electroporation.

Preclinical studies in animal models have been performed to establish IRE safety profiles and dose responses.[64,67–70] An in vitro study conducted by Rubinsky *et al*. demonstrated a total of 90 pulses at 250 V/cm for 100 μs separated by 100 ms could completely ablate prostate cancer cells without inducing thermal injury. Initial studies in human subjects are currently underway in select centers in the United States.

## HISTOTRIPSY

Histotripsy is a new transcutaneous ablative technique under development by a multidisciplinary team at the University of Michigan. Much like HIFU, histotripsy is based on the propagation of ultrasound waves through tissues, energy focusing, and subsequent conversion of energy at the ablation site. However, histotripsy differs significantly from HIFU in the method of tissue ablation. HIFU produces a thermal effect and subsequent coagulative necrosis, whereas histotripsy induces nonthermal mechanical disruption of cells. During histotripsy when acoustical intensity exceeds 1500–2000 W/cm^2^ rapid cycling from compression to rarefaction results in the formation of microbubbles in the tissue. These bubbles oscillate and violently collapse releasing tremendous amounts of energy that can fragment and subdivide tissue, resulting in cellular destruction.[71]

Histotripsy is performed by a system consisting of a high power transducer on a 3-axis computer-controlled positioning system. The piezocomposite transducer has a 145 mm diameter and 100 mm focal length, emitting ultrasound at 500–1000 kHz.[71] Pulse repetition rates range from 100 to 1000 Hz with a pulse duration of 5–20 μs and duty cycle of 0.2–0.5%.[72] The histotripsy system is acoustically coupled to the subject by a bath of degassed water. A hole in the center of the transducer permits a confocally aligned monitoring probe. Upon the initiation of energy delivery, a region of transient hyperechogenicity is visualized at the focal point probably representing a bubble cloud.[73] Electronic steering is used to direct the transducer focal point in a grid-like fashion across the targeted tissue.

Work has focused on the nature of ultrasound mechanical tissue fractionation, the effects of various acoustic parameters and the feasibility of transcutaneous treatment of normal animal renal and prostatic tissue.[71–74] Histologic examination of lesions created by histotripsy show a thin, well-demarcated rim of intact cells with a transition zone of only several cells containing pyknotic nuclei surrounding the focal zone. The focal zone contains a liquefied homogenous slurry of cellular debris without evidence of intact cells.[72,73] A recent work demonstrated the differential effects of histotripsy in a porcine renal model.[74] Targeted tissue in the cortex readily cavitates, whereas medullary tissue is more resistant (presumably due to increased fibrous elements in the tissue) and the collecting system is relatively spared.

Although still in the initial developmental stages, the unique characteristics of the energy delivered during histotripsy may make it well suited for clinical use in human subjects. Transcutaneous treatment is advantageous compared with other techniques that require percutaneous needle placement. Nonthermal ablative mechanisms obviate concerns for heat sink effects from large adjacent vessels. The low-duty cycle allows for real-time observation of the ablation process with diagnostic ultrasound between ultrasound pulses.[71] Transcutaneous ablation of renal tissue is challenging due to the difficulties with acoustic windows and constant lesion motion from respiration during treatment. Because histotripsy is nonthermal and has a low-duty cycle, it may be less susceptible to target motion than HIFU. Studies evaluating transabdominal histotripsy in a canine prostate model are encouraging and are paving the way for histotripsy in human subjects. Preliminary studies of the acoustic windows into the human pelvis have recently been performed to determine the feasibility of a transperineal transducer.[75]

## CONCLUSION

Ablative therapy, specifically RFA and CA, for the treatment of SRMs has produced encouraging intermediate term results. Further research on specific energy type, biologic effect, delivery system, and molecular adjuvants may improve oncologic results and broaden the indications for ablative therapy
